# Integrative PANoptosis-focused omics analysis uncovers GSDMC as a candidate biomarker in breast cancer

**DOI:** 10.3389/fcell.2026.1841350

**Published:** 2026-06-15

**Authors:** Jinghui Hong, Zhihao Wei, Mengxin Li, Xiaochuan Gao, Yuheng Wu, Xuyutian Wang, Xin Wang, Tianxing Zhang, Dong Song

**Affiliations:** 1 Department of Breast Surgery, General Surgery Centre, The First Hospital of Jilin University, Changchun, China; 2 Department of Population Health Sciences, Bristol Medical School, University of Bristol, Bristol, United Kingdom; 3 Department of Molecular Biology, College of Basic Medical Sciences, Jilin University, Changchun, China

**Keywords:** biomarker, breast cancer, GSDMC, PANoptosis, prognosis

## Abstract

**Background:**

PANoptosis, a form of programmed cell death involving crosstalk among pyroptosis, apoptosis, and necroptosis, has recently emerged as a key player in tumor progression and therapy resistance. Despite growing evidence linking PANoptosis to various cancers, the prognostic significance of PANoptosis-related genes (PANRGs) and their roles in breast cancer is not well defined. Investigating their relevance could reveal novel biomarkers for patient stratification and identify new therapeutic targets to overcome resistance to existing treatments.

**Methods:**

Multi-omics data from TCGA and GEO were analyzed to identify differentially expressed PANRGs. A prognostic signature was constructed via LASSO and multivariate Cox regression and validated in independent cohorts. Functional, microenvironmental, mutational, and drug sensitivity analyses were performed, with key findings validated experimentally.

**Results:**

A five-gene PANRG signature (AIFM1, GSDMC, IRF1, IL18, GZMB) effectively stratified patients into high- and low-risk groups. High-risk patients showed poorer overall survival, advanced TNM stages, immunosuppressive “cold tumor” microenvironments, metabolic reprogramming, higher TP53 mutation frequency, and increased resistance to chemotherapy but greater sensitivity to HER2/mTOR/EGFR-targeted therapies. Single-cell analysis revealed enhanced stromal communication and macrophage dynamics in high-risk patients. Experimental validation confirmed GSDMC upregulation in breast cancer tissues and its association with poor prognosis.

**Conclusion:**

This study developed and validated a novel PANoptosis-related prognostic signature for breast cancer, which predicts patient survival, immune landscape, metabolic features, and therapy response. The findings highlight the potential of PANRGs as biomarkers and therapeutic targets, providing a foundation for precision medicine in breast cancer treatment.

## 1 Introduction

Breast cancer (BC) is the predominant form of cancer in women, critically endangering their health (Chen et al., 2025). The pathogenesis of BC is remarkably complex, and the comprehensive molecular mechanisms underlying its development have not been fully elucidated, hindering the development of targeted therapies for various pathological subtypes (Xio et al., 2025). Consequently, researchers must intensify efforts to unravel the multifaceted nature of BC, particularly addressing critical challenges such as drug resistance, to develop effective treatment strategies. These endeavors are laying the foundation for the era of precision oncology in BC management.

PANoptosis represents a unique and novel form of programmed cell death (PCD) pathways that involves intricate interactions among pyroptosis, apoptosis, and necroptosis. However, its underlying mechanisms cannot be fully explained by any of these individual PCD pathways alone (Qi et al., 2023). As research into the biological processes and mechanisms of PANoptosis progresses, scientists have discovered that PANoptosis can be triggered by diverse stimuli and is particularly closely associated with cancer development (Sun et al., 2024). PANoptosis plays a pivotal yet dual role in cancer biology, contributing to tumorigenesis, progression, and therapy resistance while simultaneously exhibiting both tumor-suppressive effects via immune activation and pro-tumorigenic effects through promoting cancer cell survival and proliferation (Xiong, 2023). Current research has identified differential expression of PANoptosis-related genes (PANRGs), such as NLRP3 and TNFAIP3, across various cancer types (Gao et al., 2024). Interestingly, many PANRGs have been found to serve as risk factors for certain cancers (Gao et al., 2024).

Currently, research on PANoptosis in BC remains limited. Therefore, further investigation into the role of PANoptosis in the initiation and progression of BC, along with the development of a PANoptosis-related prognostic signature, holds significant importance for BC treatment. In this study, we aimed to explore gene signatures associated with PANoptosis through integrated analysis of independent bulk and single-cell omics datasets. We established a prognostic model based on PANRGs to predict outcomes, immune status, and treatment responses in BC patients. Additionally, we validated the core gene GSDMC using BC cell lines, patient samples, and independent patient cohorts, suggesting its potential as a promising prognostic biomarker for BC.

## 2 Materials and methods

### 2.1 Data collection and processing

We obtained transcriptomic sequencing data and clinical information (including age, survival time and status) from The Cancer Genome Atlas (TCGA) data portal (https://portal.gdc.cancer.gov/) as the training set for model development. For external validation, datasets GSE20685 and GSE58812 were acquired from the Gene Expression Omnibus (GEO) database (https://www.ncbi.nlm.nih.gov/geo/), while GSE161529 was used for single-cell RNA sequencing (scRNA-seq) analysis. The 93 PANRGs were derived from both previously published literature on PANoptosis and the Molecular Signatures Database (MSigDB) (https://www.gsea-msigdb.org/gsea/msigdb/) (Liberzon et al., 2015), where gene sets were retrieved using the keywords ‘pyroptosis’, ‘apoptosis’, and ‘necroptosis’. Detailed information on all databases is provided in Table 1.

**TABLE 1:** Clinical sample characteristics in TCGA-BRCA and GEO datasets.

Database	Sample sequencing type	Detailed information
TCGA-BRCA	RNA- sequencing	1113 cases of BC samples and 113 cases of normal tissue samples
GSE20685	RNA- sequencing	327 cases of BC samples
GSE58812	RNA- sequencing	107 cases of BC samples
GSE161529	scRNA-seq	34 cases of BC samples and 13 cases of normal tissue samples

### 2.2 Development and validation of a PANRGs prognostic signature

Differentially expressed genes (DEGs) between BC and normal tissues were identified using the “DESeq2” R package, with thresholds set at |log2-fold change (FC)| > 0.584 and an adjusted P < 0.05 (FDR correction), and visualize the top DEGs by generating a volcano plot with “ggplot2” R package and a heatmap with “pheatmap” R package. Subsequently, we identified PANRGs by intersecting the datasets using the “VennDiagram” R package. Chromosomal localization of these DEGs was analyzed based on the GENCODE database (https://www.gencodegenes.org) and visualized using the “rcircos” R package. Using the Gene Set Variation Analysis (GSVA) algorithm, we computed a PANoptosis score for each tumor sample in the TCGA-BRCA cohort by performing gene set enrichment analysis (GSEA)with the PANRGs set. The correlation between this score and DEGs expression levels was further evaluated. Functional enrichment analysis, including Gene Ontology (GO) and Kyoto Encyclopedia of Genes and Genomes (KEGG) pathway analyses, was then performed using the “clusterProfiler” R package with a significance threshold of adjusted P < 0.05. To explore protein-protein interactions (PPIs) among these genes, we queried the STRING database (v12.0, http://www.string-db.org/) using the identified PANRGs (Szklarczyk et al., 2023).

Univariate Cox regression analysis was performed to screen for PANRGs significantly associated with patient prognosis. To enhance model stability, we applied Least Absolute Shrinkage and Selection Operator (LASSO) regression via the “glmnet” R package. The optimal regularization parameter λ was determined by 10-fold cross-validation, and the model with lambda. min = 0.0055 was selected for subsequent analysis. Using multivariate Cox regression, we constructed a prognostic model based on the selected PANRGs and derived a PANoptosis-related risk score using the following formula:

$$\text{Risk Score}=\left(\text{gene}1\times \text{coefficient}1\right)+\left(\text{gene}2\times \text{coefficient}2\right)+\ldots +\left(\text{geneN}\times \text{coefficientN}\right)$$

Patients were stratified into high- and low-risk groups based on the median PANoptosis-related risk score. Time-dependent receiver operating characteristic (ROC) analysis performed using the “timeROC” R package, was employed to evaluate the predictive accuracy of the PANRGs signature. Additionally, the diagnostic performance of prognostic genes in distinguishing between tumor and normal tissues was evaluated using the “pROC” R package, with accuracy measured by the area under the curve (AUC) value.

### 2.3 Risk score-clinical characteristics correlation analysis

Using TCGA-BRCA patient data, we compared the distribution differences of risk scores across various clinical characteristics, including age and TNM staging. The results were visualized using grouped stacked bar plots generated with the “ggplot2” and “ggpubr” R packages. Subsequently, we performed chi-square tests to examine the association between risk stratification (high/low) and different clinical features by comparing patient numbers and proportions across groups. The correlation results were further visualized as a heatmap using the “ComplexHeatmap” R package.

### 2.4 Construction and evaluation of the nomogram

We integrated risk scores with clinical characteristic parameters and performed both univariate and multivariate Cox proportional hazards regression analyses. Using the “survival” R package, we constructed forest plots to visualize statistically significant indicators (P < 0.05). Significant independent prognostic factors identified through multivariate analysis were subsequently incorporated into a nomogram model using the “rms” and “regplot” R packages. Finally, we conducted a comprehensive evaluation of the nomogram’s predictive performance through ROC analysis, calibration plots, and decision curve analysis (DCA) to validate its clinical utility for prognostic assessment in BC.

### 2.5 GSEA analysis

GSEA analysis was performed based on risk score stratification of TCGA-BRCA patients, using KEGG gene sets from the MsigDB database to assess pathway enrichment. Significant pathways were filtered with the criteria |NES| > 1, P < 0.05, and FDR < 0.25. The top significantly enriched pathways in the high- and low-risk groups were extracted and visualized using the “GseaVis” R package.

### 2.6 Analysis of correlation with immune status

Based on the “ESTIMATE” R package, we calculated the stromal score and immune score in tumor tissues for high- and low-risk groups of TCGA-BRCA patient samples. These scores were then integrated to derive the Estimation of STromal and Immune cells in MAlignant Tumor tissues using Expression data (ESTIMATE) score and tumor purity. Next, using the Cell-type Identification By Estimating Relative Subsets Of RNA Transcripts (CIBERSORT) algorithm from the “IOBR” R package, we quantified the infiltration levels of 22 immune cell subtypes in the high- and low-risk groups. After excluding samples without statistically significant differences, we compared immune cell infiltration patterns and analyzed the correlation between PANRGs and the infiltration levels of various immune cells. Finally, we employed the Tumor Immune Dysfunction and Exclusion (TIDE) (http://tide.dfci.harvard.edu) to predict the potential response of high- and low-risk groups to immune checkpoint inhibitor therapy (Jiang et al., 2018).

### 2.7 Tumor mutation analysis

Somatic mutation data for TCGA-BRCA was retrieved via the “TCGAbiolinks” R package, followed by data quality control and standardization using the “maftools” R package, including the removal of duplicate samples and functional annotation of mutation sites. Subsequently, we identified the top 20 most frequently mutated genes across all samples and compared their mutation frequencies as well as associated mutational signatures between different risk subgroups using waterfall plots.

### 2.8 Analysis of sensitivity and potential drugs in BC

To further investigate the predictive potential of the risk prognostic signature in assessing drug sensitivity for BC treatment, we utilized the “pRRophetic” R package to predict the half-maximal inhibitory concentration (IC50) values of various drugs. Subsequently, we compared the sensitivity of clinically commonly used BC drugs between high- and low-risk groups, with results visualized using boxplots generated by the “ggplot2” R package. Additionally, we integrated transcriptomic data from cell lines and drug sensitivity profiles from the Cancer Therapeutics Response Portal (CTRP) (http://portals.broadinstitute.org/ctrp/) and the Profiling Relative Inhibition Simultaneously in Mixtures (PRISM) database (https://www.theprismlab.org/) to predict the therapeutic response of clinical samples to standard BC drugs (Rees et al., 2016). Using the area under the drug concentration-response curve as an evaluation metric, we calculated Spearman correlation coefficients between predicted AUC values and patient risk scores. Correlation scatter plots and drug sensitivity boxplots were constructed using the “ggplot2” R package, with statistical significance levels annotated on the boxplots.

### 2.9 Analysis of scRNA-seq datasets

This study performed integrated analysis of scRNA-seq data from tumor and normal tissue samples in the GSE161529 dataset using Seurat (v5). After batch effect correction with BBKNN algorithm, dimensionality reduction and clustering were conducted through PCA and UMAP. Cell type annotation was accomplished based on cell population-specific marker genes, with malignant epithelial cells identified using plasma cells as reference. The inferCNV algorithm was employed to evaluate chromosomal copy number variation (CNV) patterns: single-cell gene expression data were first Z-score normalized, followed by quantification of CNV magnitude through calculating the sum of squared scaled expression values across all genes. Tumor cells were stratified into high- and low-risk groups based on median-dichotomized average risk scores calculated from key gene coefficients, with intergroup cell communication differences analyzed using the “CellChat” R package. For the PANoptosis gene set, UCell algorithm was applied to compute apoptosis enrichment scores across cell types, defining the highest-scoring population as pivotal cells. Subsequent reclustering of pivotal cells with CytoTRACE scoring assessed their differentiation potential. Finally, pseudotemporal trajectories were constructed via “monocle2” R package to elucidate differentiation dynamics and key gene expression patterns in pivotal cell subpopulations.

### 2.10 Cell culture

The human breast epithelial cell line MCF-10A and nine human BC cell lines (MCF-7, MDA-MB-231, MDA-MB-468, MDA-MB-453, MDA-MB-436, HCC1937, HCC38, HCC1806, BT549, SKBR3, and AU565) were obtained from the American Type Culture Collection (ATCC). All BC cell lines were maintained in a humidified incubator at 37 °C with 5% CO2, cultured in either high-glucose DMEM medium (Hyclone) or 1640 medium (Hyclone) supplemented with 10% fetal bovine serum (CellMax) and 1% penicillin/streptomycin (Beyotime Biotechnology). The MCF-10A cells were cultured in MCF-10A-specific growth medium (Procell).

### 2.11 Patient sample collection

All procedures involving human samples in this study were conducted in accordance with the principles of the Declaration of Helsinki. The study protocol was approved by the Ethics Committee of the First Hospital of Jilin University (2023-727) (Hong et al., 2023). Paired tissue samples (BC and adjacent normal tissues) were collected from 10 BC patients. In addition, paraffin-embedded tissue sections from 41 patients who underwent surgical resection in January 2020 and had complete follow-up records were also included in the study.

### 2.12 Quantitative RT-PCR (qRT-PCR) in cell lines and breast tissues

Total RNA was extracted and reverse-transcribed into cDNA, followed by qRT-PCR analysis of five PANRGs transcripts using 2× RealStar Fast SYBR qPCR Mix (GenStar, Beijing, China). All oligonucleotide primers targeting AIFM1, GSDMC, IL18, IRF1, GZMB, and the reference gene GAPDH were commercially synthesized by Jilin Comate Bioscience Co., Ltd., (Jilin, China). The specific primer sequences were as follows: AIFM1 (F: AAG​TCA​GAC​GAG​AGG​GGG​TTA; R: GCC​AAC​TCA​ACA​TTG​GGC​T), GSDMC (F: TCC​ATG​TTG​GAA​CGC​ATT​AGC; R: CAA​ACT​GAC​GTA​ATT​TGG​TGG​C), IL18 (F: TCT​TCA​TTG​ACC​AAG​GAA​ATC​GG; R: TCC​GGG​GTG​CAT​TAT​CTC​TAC), IRF1 (F: ATG​CCC​ATC​ACT​CGG​ATG​C; R: CCC​TGC​TTT​GTA​TCG​GCC​TG), GZMB (F: CCC​TGG​GAA​AAC​ACT​CAC​ACA; R: GCA​CAA​CTC​AAT​GGT​ACT​GTC​G), and GAPDH (F: GGA​GCG​AGA​TCC​CTC​CAA​AAT; R: GGC​TGT​TGT​CAT​ACT​TCT​CAT​GG). Target gene expression levels were normalized against the internal reference gene GAPDH for quantitative analysis.

### 2.13 Western blot

Total protein was extracted from tissues or cells using RIPA lysis buffer, and the protein concentration was determined with a BCA assay kit (Beyotime, China). Equal amounts of protein samples were separated by SDS-PAGE and subsequently transferred onto a PVDF membrane via the wet transfer method. The membrane was blocked with 5% skim milk at room temperature for 1 h, followed by incubation with specific primary antibodies and an HRP-conjugated secondary antibody. Finally, protein bands were visualized using an ECL chemiluminescence reagent (Beyotime, China) and imaged with a gel documentation system. The following antibodies were used: GSDMC (Proteintech, Cat No. 30469-1-AP; suitable for immunohistochemistry) and Actin (Proteintech, Cat No. 66009-1-Ig).

### 2.14 Immunohistochemistry and H-score assessment

Tissue sections (5 μm) were prepared from 4% paraformaldehyde-fixed, paraffin-embedded blocks. After deparaffinization and rehydration through an ethanol series, endogenous peroxidase activity was blocked using 3% hydrogen peroxide. The sections were then incubated with normal goat serum to reduce nonspecific binding. This was followed by sequential incubation with primary and secondary antibodies. Finally, the sections were mounted and observed under a light microscope. All slides were independently evaluated by two pathologists in a blinded manner. Positive staining was assessed based on staining intensity and the percentage of positive cells. Staining intensity was categorized as follows: negative (0), weak (1+), moderate (2+), or strong (3+). The H-score was calculated by multiplying the staining intensity by the average percentage of positive cells per field of view, resulting in a score ranging from 0 to 300.

### 2.15 Statistical analysis

All statistical analyses and visualizations were performed using R software (version 4.4.0). The significance threshold was set at P < 0.05, with P < 0.0001, P < 0.001, P < 0.01, and P < 0.05 denoted as “****”, “***”, “**” and “*”, respectively. Additional statistical comparisons, including two-tailed unpaired t-tests, one-way ANOVA, and two-way ANOVA, were conducted using GraphPad Prism (version 9.0.0).

## 3 Result

### 3.1 Screening and identification of DEGs related to PANoptosis

**FIGURE 1:**
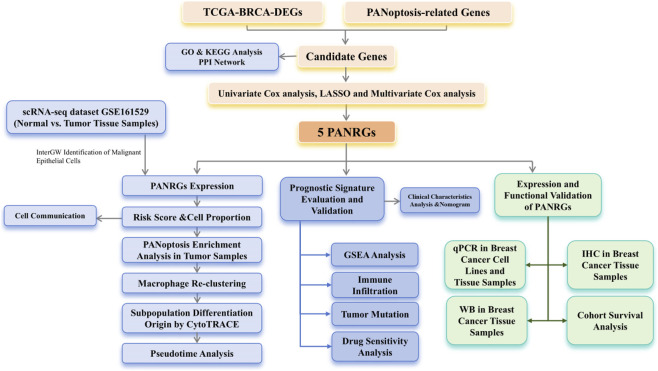
Study workflow overview.

**FIGURE 2:**
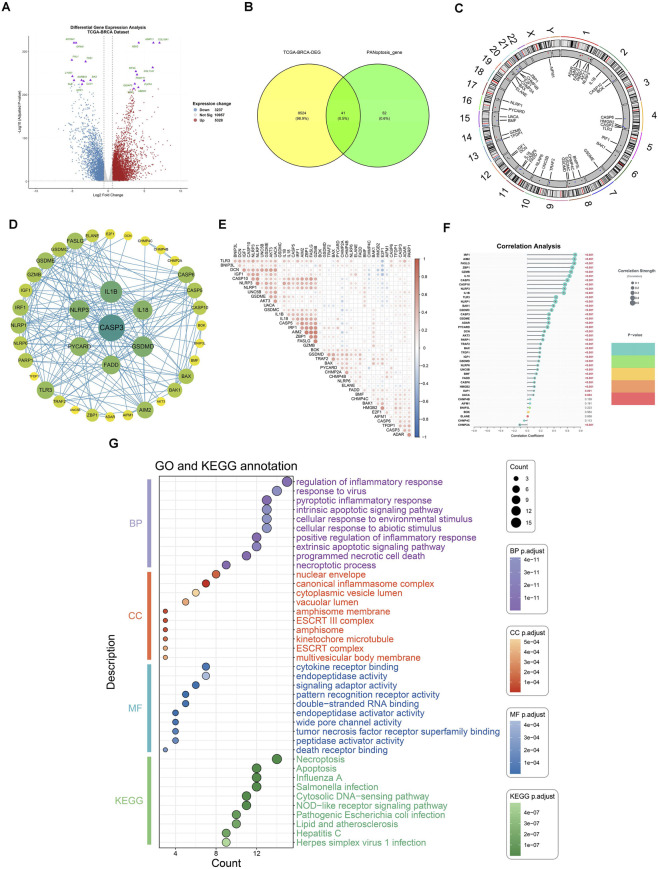
Expression differences and enrichment analysis of PANRGs in the TCGA-BRCA cohort. **(A)** Volcano plot of DEGs in the TCGA-BRCA cohort; **(B)** Venn diagram illustrating the overlap between RANRGs and DEGs; **(C)** Chromosomal localization map of 41 PANRGs; **(D)** PPI network analysis of key DEGs; **(E)** Co-expression network of key DEGs;** (F)** The correlation between the expression of DEGs and the PANoptosis score; **(G)** GO and KEGG enrichment analysis of key DEGs.

Figure 1 presents the workflow of this study. Initially, we identified 8553 DEGs in the TCGA-BRCA dataset, comprising 5328 upregulated and 3227 downregulated genes (Figure 2A; Supplementary Figure S1). By intersecting these TCGA-BRCA DEGs with the PANoptosis gene set, we obtained 41 candidate DEGs associated with PANRGs (Figure 2B). Chromosomal locations of these 41 PANRGs were mapped according to the GENCODE database (Figure 2C). Subsequent PPI network analysis revealed that key DEGs including IL1B, IL18, NLRP3, CASP3, PYCARD, GSDMD, and FADD formed a complex interaction network (Figure 2D), demonstrating extensive interconnections among multiple genes (Figure 2E). Additionally, the expression of DEGs showed a strong correlation with the PANoptosis score (Figure 2F).

Given the diverse biology of PANoptosis, which encompasses tumor-intrinsic cell death mechanisms as well as immune and inflammatory responses, we proceeded to perform enrichment analyses of these DEGs. GO analysis showed these genes were primarily enriched in biological processes (BP) such as regulation of inflammatory response, pyroptotic inflammatory response, necroptotic process, and intrinsic/extrinsic apoptotic signaling pathway. For cellular components (CC), significant enrichment was observed in canonical inflammasome complex, ESCRT complex and multivesicular body membrane. Molecular function (MF) analysis indicated involvement in pattern recognition receptor activity, death receptor binding and endopeptidase activator activity (Figure 2G). KEGG pathway analysis demonstrated these genes were mainly associated with Necroptosis, Apoptosis, NOD-like receptor signaling pathway and Cytosolic DNA-sensing pathway (Figure 2G). In summary, the enrichment analysis results suggest these candidate DEGs show significant associations with inflammatory responses and immune-related functions.

### 3.2 Construction and validation of PANRGs prognostic signature

**FIGURE 3:**
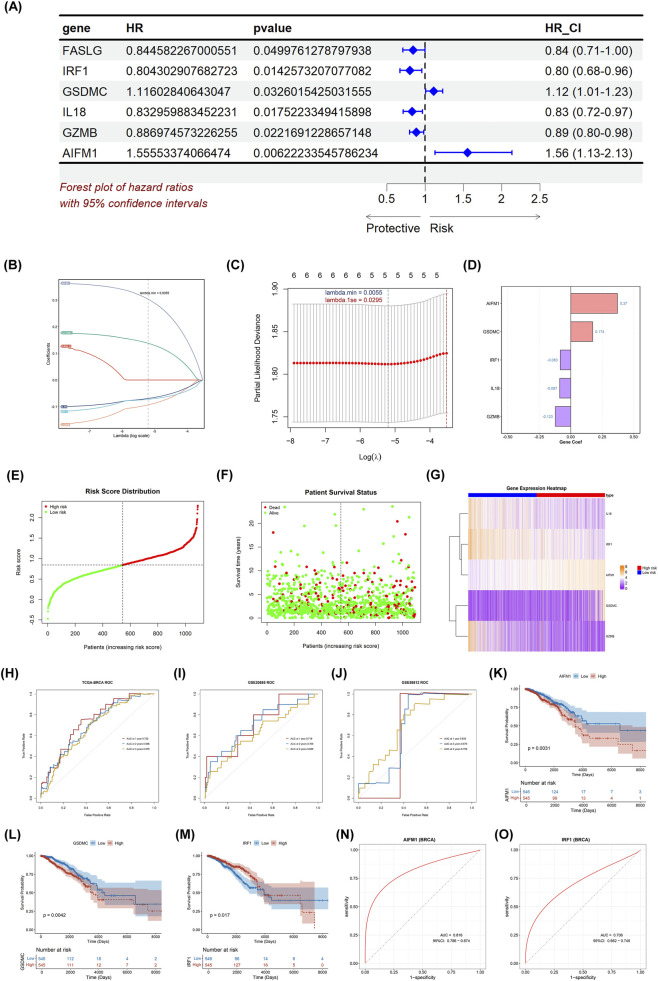
Construction and validation of risk signature for BC patients based on the TCGA-BRCA database. **(A)** Univariate Cox regression analysis of PANRGs; **(B)** LASSO regression analysis of PANRGs; **(C)** Cross-validation in LASSO regression; **(D)** Contribution coefficients of the 5 identified PANRGs; **(E)** Distribution of PANRG-based risk scores; **(F)** OS status distribution across risk groups; **(G)** Expression patterns of the 5 PANRGs in different risk groups; **(H-J)** AUC values of ROC curves for risk scores (TCGA-BRCA cohort, GSE20685 and GSE55812 validation set);** (K–M)** Kaplan-Meier overall survival curves comparing groups based on the expression of the PANRGs; **(N–O)** ROC curve analysis of PANRGs for discriminating between tumor and normal tissues.

Univariate Cox regression analysis of 41 key DEGs identified 6 PANRGs significantly associated with prognosis (P < 0.05) (Figure 3A). Subsequent LASSO regression analysis for feature selection (Figure 3B) was performed, with the optimal regularization parameter (λ = 0.0055) determined through 10-fold cross-validation (Figure 3C). Multivariate Cox regression ultimately retained 5 PANRGs: AIFM1, GSDMC, IRF1, IL18 and GZMB (Figure 3D). Using the expression levels and regression coefficients of these 5 PANRGs, we calculated a risk score for each patient as follows: RiskScore = (0.37 × AIFM1) + (0.174 × GSDMC) + (−0.083 × IRF1) + (−0.087 × IL18) + (−0.123 × GZMB). Patients were then stratified into high- and low-risk groups based on the median risk score. The risk score distribution showed an increasing trend from low to high risk (Figure 3E). Higher risk scores correlated with increased mortality risk and shorter overall survival (OS) (Figure 3F). Expression analysis revealed that AIFM1 and GSDMC were highly expressed in the high-risk group, while IRF1, IL18, and GZMB showed higher expression in the low-risk group (Figure 3G). In the TCGA-BRCA training set, the AUC values for 1-, 2-, and 3-year survival were 0.732, 0.696, and 0.675, respectively (Figure 3H). Validation in the GSE20685 dataset yielded AUC values of 0.719, 0.709, and 0.629 (Figure 3I), while the GSE55812 dataset showed AUC values of 0.635, 0.679, and 0.706 (Figure 3J). All AUC values exceeded 0.6 across different time points, demonstrating the signature‘s robust predictive capability for short-to medium-term prognosis. Furthermore, most PANRGs were significantly associated with prognosis (Figures 3K–M; Supplementary Figures S2A,B), and the majority also demonstrated consistent diagnostic efficacy between tumor and normal tissues (Figures 3N,O; Supplementary Figures S2C–E).

Similar patterns were observed in the validation sets (GSE20685 and GSE55812), with risk score distribution plots showing increasing scores and survival status scatter plots indicating more concentrated death events in high-risk groups (Supplementary Figures S2F–I). Gene expression heatmaps in the validation sets consistently showed higher expression of AIFM1 and GSDMC in high-risk groups, mirroring the training set results (Supplementary Figures S2J,K). Kaplan-Meier survival analysis confirmed significantly worse OS in high-risk patients (TCGA-BRCA: P = 2e-04; GSE20685: P = 0.03; GSE55812: P = 0.034) (Supplementary Figures S2L–N). Together, these collective results demonstrate the robustness of our prognostic signature.

### 3.3 Evaluation clinicopathologic nomogram

**FIGURE 4:**
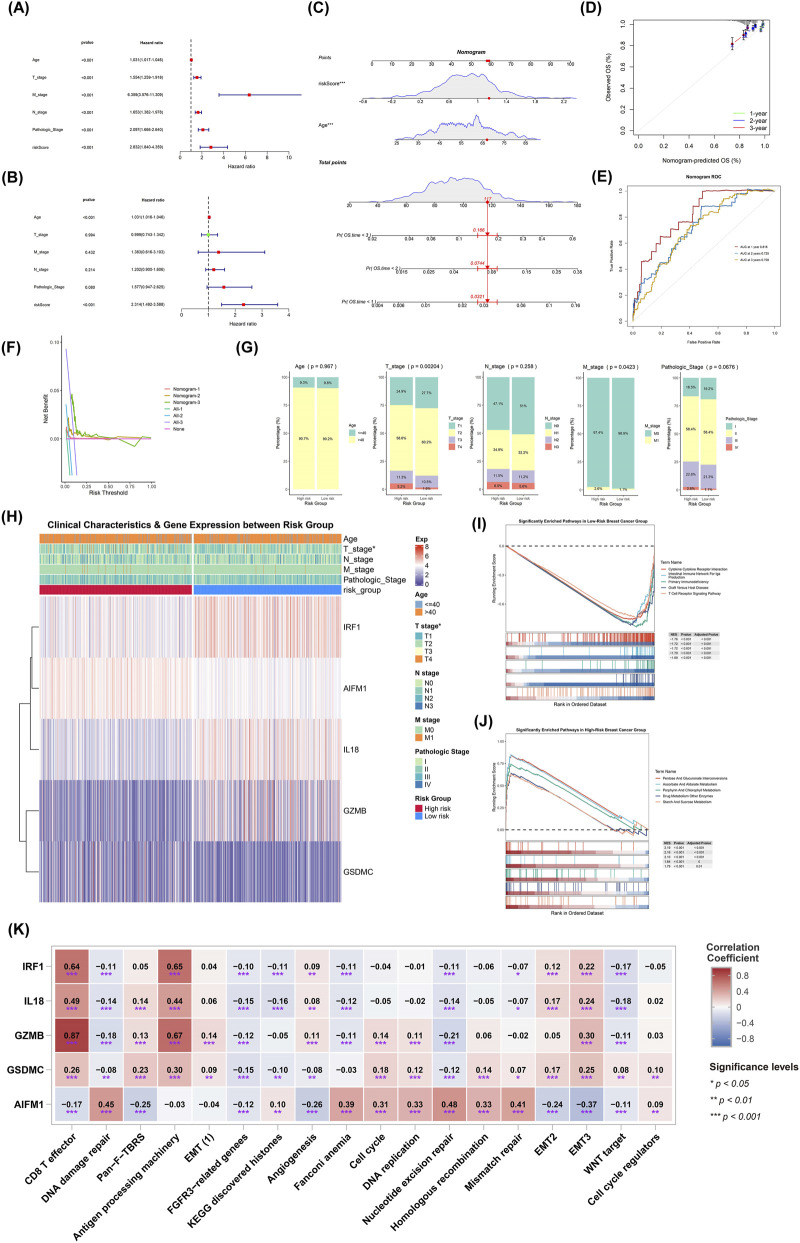
A prognostic nomogram's value and its clinical and molecular correlations. **(A)** Univariate Cox analysis of risk score for OS in the TCGA-BRCA cohort;** (B)** Multivariate Cox regression analysis of risk score for OS in the TCGA-BRCA cohort;** (C)** Nomogram prediction model; **(D)** Calibration curve of the nomogram prediction model;** (E)** ROC curve of the nomogram prediction model; **(F)** DCA of the nomogram prediction model; **(G)** Differences in risk distribution between high-risk and low-risk groups across various clinical characteristics; **(H)** Heatmap showing the expression profiles of PANRGs in different risk groups stratified by clinical features; **(I)** GSEA of the low-risk group; **(J)** GSEA of the high-risk group; **(K)** Correlations between 5 PANRGs and known core biological pathway scores.

To validate the prognostic value of the risk score as an independent indicator for BC, we conducted a systematic Cox regression analysis in the TCGA-BRCA cohort. Univariate Cox analysis demonstrated that age, TNM stage, pathological stage and risk score were all significantly associated with overall survival (P < 0.001, Figure 4A). Multivariate Cox analysis further confirmed that both age and risk score served as independent predictive factors (P < 0.001, Figure 4B). Based on these findings, we developed a nomogram prediction model integrating the risk score and age (Figure 4C), which indicated a 16.6% probability of death within 3 years when the total score reached 117 points.

Model validation revealed the following results: The calibration curve demonstrated good accuracy for 1- to 3-year predictions (Figure 4D); time-dependent ROC analysis showed AUC values of 0.816 (1 year), 0.725 (2 years), and 0.708 (3 years) (Figure 4E); These values were consistently higher than those of any individual clinical variable or the risk score alone, demonstrating optimal overall predictive performance (Supplementary Figures S3A–B). DCA confirmed the model’s clinical utility across all time points, with particularly robust performance in 3-year predictions (Figure 4F). This nomogram model provides a reliable tool for precise prognostic assessment in BC patients.

### 3.4 Correlation between risk score and clinical characteristics with pathway enrichment analysis

To evaluate the clinical relevance of the risk score model, we systematically analyzed the distribution differences in risk scores between high- and low-risk groups across various clinical characteristics. Results revealed significant differences between the two groups in T stage (P = 0.00204) and M stage (P = 0.0423) (Figure 4G). Specifically, the proportions of patients with T3–T4 stage and M1 stage were significantly higher in the high-risk group than in the low-risk group. However, no significant differences were observed in age (P = 0.967), N stage (P = 0.258), or pathological stage (P = 0.0676) between the two groups. Further visualization via heatmap analysis demonstrated distinct patterns in T-stage distribution and key gene expression profiles between the high- and low-risk groups (Figure 4H). These findings not only validate the clinical relevance of the risk score model but also highlight the close association between key gene expression patterns and clinicopathological features as well as prognostic risk.

To further elucidate the molecular mechanisms underlying the differences between the high- and low-risk groups, we performed GSEA analysis. The results showed that the low-risk group was significantly enriched in immune-related pathways, including cytokine-cytokine receptor interaction (NES = −1.76, P < 0.001) and primary immunodeficiency (NES = −1.72, P < 0.001) (Figure 4I). In contrast, the high-risk group exhibited marked activation of multiple metabolic pathways, particularly pentose and glucuronate interconversions (NES = 2.19, P < 0.001) and ascorbate and aldarate metabolism (NES = 2.16, P < 0.001) (Figure 4J). These findings suggest that patients in the low-risk group may benefit from an active tumor microenvironment (TME), whereas the high-risk group displays prominent metabolic reprogramming, which may serve as a critical molecular basis for their poor prognosis. Additionally, analysis of a core set of biologically relevant pathways closely associated with tumor immunity, as curated from the study by Mariathasan et al. (2018), revealed that five PANRGs may be implicated in immune-related biological processes such as CD8^+^ T cell effector function and antigen processing (Figure 4K).

### 3.5 Association between immune infiltration and risk score of PANRGs

**FIGURE 5:**
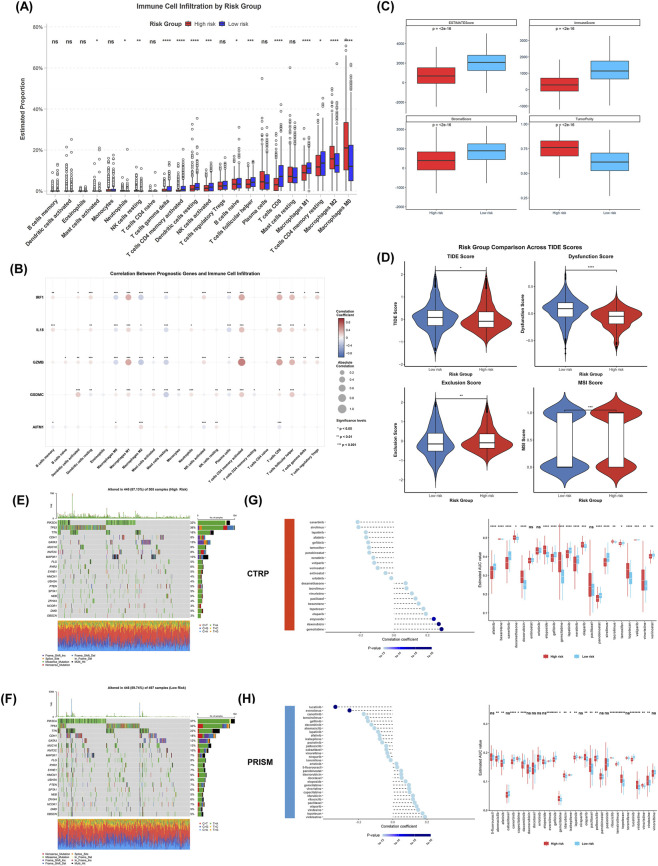
Integrating PANRGs signature with immune infiltration, tumor mutations, and drug sensitivity in BC; **(A)** Immune infiltration analysis using CIBERSORT. **(B)** Correlation analysis between PANRGs and immune cells;** (C)** ESTIMATE analysis revealed lower immune infiltration scores in the high-risk group; **(D)** TIDE analysis predicting tumor immune dysfunction and exclusion; **(E)** Somatic mutation profiles of high-risk group patients in the TCGA-BRCA cohort; **(F)** Somatic mutation profiles of low-risk group patients in the TCGA-BRCA cohort; **(G)** Differential drug responses to common therapeutic agents between risk groups using the CTRP database; **(H)** Evaluation of treatment response variations to frequently used drugs in high- and low-risk groups based on PRISM database analysis.

To investigate the relationship between risk scores and immune infiltration, we performed a comprehensive multi-algorithm analysis. CIBERSORT analysis revealed significant differences in immune cell composition between high- and low-risk groups (Figure 5A; Supplementary Figure S4). The high-risk group exhibited characteristic immunosuppressive features, including elevated proportions of pro-tumor M0/M2 macrophages and reduced proportions of anti-tumor immune cells such as M1 macrophages, activated CD4^+^ T cells, activated NK cells, and CD8^+^ T cells. Correlation analysis demonstrated that IRF1 and GZMB genes showed significant positive correlations (r > 0.3) with effector immune cells (CD4^+^ T cells, CD8^+^ T cells, and M1 macrophages), suggesting their potential role in activating anti-tumor immune responses. Although AIFM1, GSDMC, and IL18 were significantly associated with immune cells (p < 0.05), their correlations were relatively weak (r < 0.3; Figure 5B).

Integrated multi-algorithm analysis further highlighted distinct TME characteristics between the two risk groups. ESTIMATE analysis indicated that the high-risk group displayed a “cold tumor” phenotype, characterized by low immune/stromal scores and high tumor purity, whereas the low-risk group exhibited a “hot tumor” phenotype with abundant immune infiltration but concurrent T cell exhaustion (Figure 5C). TIDE analysis further corroborated these findings: the low-risk group had higher TIDE scores but lower immune exclusion, along with elevated microsatellite instability (MSI) scores (Figure 5D). In contrast, the high-risk group, despite relatively preserved T cell function, showed pronounced immune exclusion and overall insufficient immune infiltration. These results provide multi-dimensional insights into the biological basis of risk stratification, offering a theoretical foundation for the clinical application of our model.

### 3.6 Analysis of tumor mutation

We further compared the somatic mutational profiles between high- and low-risk groups in the TCGA-BRCA cohort. The results revealed comparable overall mutation rates between the two groups (low-risk: 89.74%, 446 out of 497 cases; high-risk: 87.13%, 440 out of 505 cases) (Figures 5E,F). Among the top 20 most frequently mutated genes, PIK3CA, TP53, and TTN ranked as the top three, with missense mutations being the predominant alteration type. Notably, TP53 mutations were more prevalent in the high-risk group (36% vs. 32%), whereas TTN (22% vs. 16%), PIK3CA (37% vs. 32%), and CDH1 (18% vs. 8%) exhibited higher mutation frequencies in the low-risk group. Furthermore, the low-risk group displayed a higher peak tumor mutational burden (TMB), consistent with its enhanced immunogenic characteristics, aligning with our previous immune infiltration analysis.

### 3.7 Drug sensitivity analysis based on PANRGs reveals novel therapeutic strategies for BC

By integrating multiple drug sensitivity databases, we systematically evaluated the differential responses to commonly used therapeutic agents among BC patients stratified into different risk groups. Analysis of the GDSC database revealed that among 33 commonly administered BC drugs, as many as 30 exhibited significant sensitivity differences between high- and low-risk groups (Supplementary Figure S5). Notably, patients in the high-risk group demonstrated higher IC50 values for the majority of drugs, suggesting a general trend of drug resistance. These findings were further validated in the CTRP and PRISM databases: CTRP data indicated that traditional chemotherapeutic agents (e.g., gemcitabine, doxorubicin) showed a positive correlation with risk scores, whereas targeted therapies (e.g., canertinib, sirolimus) exhibited a negative correlation (Figure 5G). PRISM data further confirmed that HER2/mTOR inhibitors (e.g., tucatinib, everolimus) were significantly negatively correlated with risk scores, while chemotherapeutic agents (e.g., vincristine) maintained a positive correlation (Figure 5H).

In-depth analysis revealed a distinctive “dual-mode” response pattern in high-risk patients: significantly increased AUC values (P < 0.05) for traditional chemotherapeutic drugs, indicating enhanced resistance, alongside significantly decreased AUC values (P < 0.05) for targeted therapies, particularly HER2/mTOR/EGFR inhibitors, suggesting markedly improved sensitivity. These findings provide critical insights for clinical personalized treatment, suggesting that high-risk patients may benefit more from targeted therapies, whereas low-risk patients may respond better to conventional chemotherapy.

### 3.8 scRNA-seq reveals cellular heterogeneity within the TME

**FIGURE 6:**
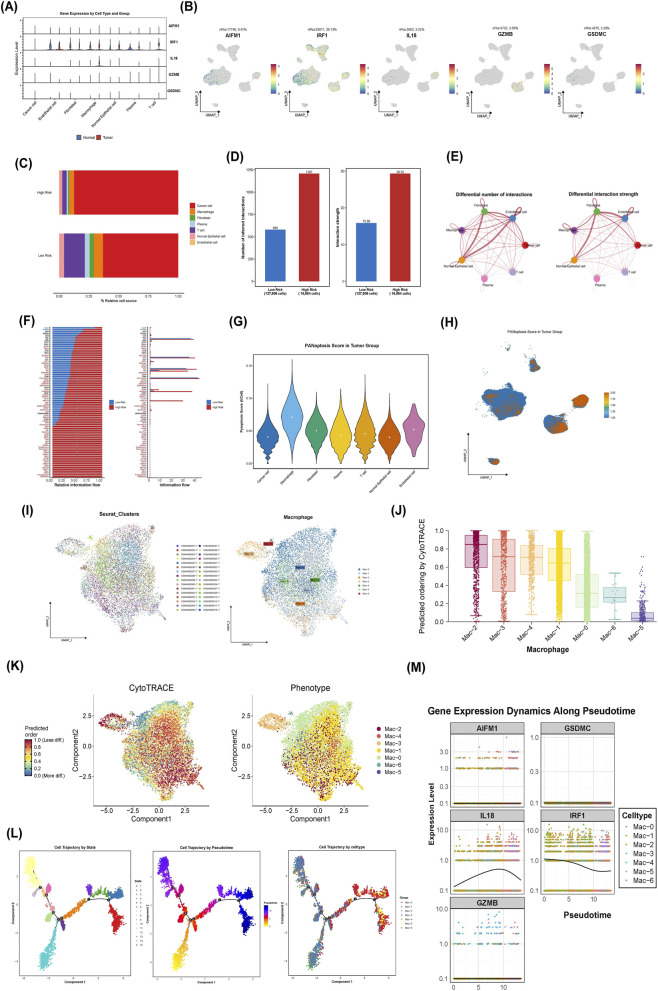
Expression of 5 PANRGs stratifies risk and maps macrophage trajectories in scRNA-seq. **(A)** Expression patterns of 5 PANRGs across cell types in the BC microenvironment; **(B)** UMAP visualization of single-cell expression distribution for the 5 PANRGs; **(C)** Proportional differences in cell types between high- and low-risk groups; **(D)** Comparative assessment of number of inferred interactions and interaction strength; **(E)** Cell-cell communication network analysis; **(F)** Comparative analysis of information flow between risk groups; **(G)** PANoptosis genes enrichment analysis; **(H)** UMAP visualization of PANoptosis genes enrichment in macrophages; **(I)** UMAP plot of reclustered macrophages (7 subpopulations); **(J)** CytoTRACE scoring indicating Mac-2 as the subpopulation with the highest differentiation potential; **(K)** Spatial distribution and differentiation status of macrophage subpopulations; **(L)** Pseudotime trajectory analysis revealing a 15-stage macrophage differentiation continuum; **(M)** Dynamic expression patterns of 5 PANRGs during macrophage differentiation.

To systematically characterize the expression patterns of risk score model-related genes in the TME, we performed integrated analysis of the multi-sample scRNA-seq dataset GSE161529. Supplementary Figures S6A,B demonstrated the robust integration consistency and reliability across multiple samples, while Supplementary Figure S6C visually presented the cellular composition of distinct clustered subpopulations. Through canonical marker-based annotation (Supplementary Figure S6D) and inferCNV algorithm application, we successfully discriminated normal from malignant epithelial cells. Supplementary analyses revealed: well-integrated epithelial subpopulations (Supplementary Figure S6E), 37 clearly-defined subclusters after re-clustering (Supplementary Figure S6F), and effective exclusion of contaminating cell types via bubble plot visualization (Supplementary Figure S6G). CNV analysis identified subclusters 2,5,12,14,17,27 and 31 exhibiting plasma cell-like patterns (Supplementary Figure S6H), with quantitative confirmation of significantly lower chromosomal copy numbers in these subpopulations (Supplementary Figure S6I), thereby classifying them as normal epithelial cells versus the remaining malignant counterparts (Supplementary Figure S6J). Expression profiling of key genes demonstrated: AIFM1 was specifically enriched in cancer cells compared to other genes; IRF1 was ubiquitously expressed across various cell types; IL18 was markedly upregulated exclusively in macrophages; GZMB exhibited selective expression in T cells; while GSDMC maintained consistently low expression levels across all cell types, though its expression in tumor cells remained higher than that in normal epithelial cells (Figure 6A; Supplementary Figures S6K–O). UMAP visualization in Figure 6B further corroborated the cell type-specific expression patterns of these five genes.

Using expression levels and contribution coefficients of five PANRGs, we calculated risk scores for individual cells in tumor samples and stratified them into high- and low-risk groups based on the median score. Cellular composition analysis revealed significantly elevated tumor cell proportions in the high-risk group, whereas the low-risk group was predominantly characterized by T cells, plasma cells, macrophages, fibroblasts, and endothelial cells (Figure 6C). Cell-cell communication network analysis demonstrated that despite their numerical minority, high-risk cells exhibited markedly enhanced interactions, manifested by increased number of inferred interactions and interaction strength (Figure 6D). Notably, fibroblast-endothelial cell interactions showed quantitative and qualitative augmentation (Figure 6E). Comparative analysis of information flow intensity identified significantly elevated activity of TENASCIN, Prostaglandin, TGFβ, and VCAM factors in the high-risk group, indicating hyperactive communication networks associated with tumor progression, angiogenesis, and immune regulation (Figure 6F).

PANoptosis genes enrichment analysis pinpointed macrophages with the highest enrichment score (Figure 6G), corroborated by UMAP visualization (Figure 6H). Subsequent reclustering subdivided macrophages into seven distinct subpopulations (Figure 6I). CytoTRACE scoring identified Mac-2 as the subpopulation with highest differentiation potential (Figure 6J), with spatial distribution analysis confirming its localization in high-potency regions (Figure 6K). Pseudotime trajectory analysis using Mac-2 as the differentiation origin reconstructed a 15-stage developmental continuum (Figure 6L). Dynamic expression profiling revealed: IL18 displayed a biphasic (net-increasing) pattern, IRF1 exhibited progressive downregulation, while AIFM1, GSDMC, and GZMB maintained low baseline expression without significant fluctuations (Figure 6M).

### 3.9 Experimental validation of PANRGs expression and clinical significance

**FIGURE 7:**
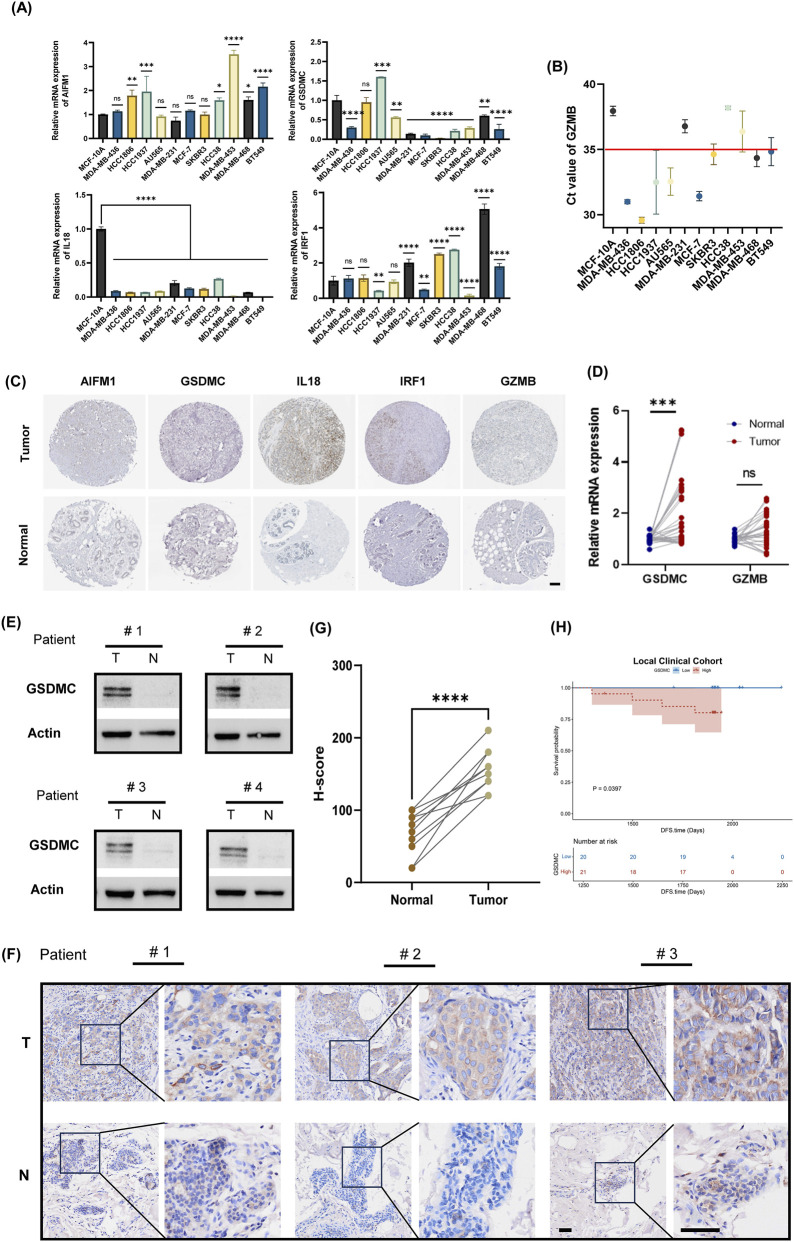
Expression and validation of PANRGs. **(A)** The mRNA expressions of AIFM1, GSDMC, IL18 and IRF1 in BC cell lines by qRT-PCR. **** P <0.0001, *** P <0.001, ** P <0.01, * P <0.05; **(B)** The Ct value of GZMB in BC cell lines by qRT-PCR; **(C)** The immunohistochemical representative images show the expression of 5 PANRG proteins in normal tissue and BC tissue, with data sourced from the HPA database. Scale Bar: 200um; **(D)** The mRNA expressions of GSDMC in Paired BC patients; **(E)** Representative western blot images of GSDMC in paired BC patients; **(F)** Representative immunohistochemistry images of GSDMC in paired BC patients, Bar: 50um; **(G)** The H-scores of GSDMC immunohistochemistry evaluated in paired BC patients; **(H)** DFS curves of BC patients with high and low GSDMC expression.

To further validate the expression patterns of PANRGs, we performed experimental verification using qRT-PCR, Western blot and IHC. First, we compared gene expression between various commonly used BC cell lines and normal breast epithelial cell lines. The results revealed that AIFM1 and IRF1 tended to be highly expressed in most BC cell lines, whereas IL18 was generally expressed at low levels. GSDMC was only expressed in a small subset of BC cell lines (Figure 7A). Notably, GZMB was not detected in the majority of the cell lines (Figure 7B). Immunohistochemical images of the five PANRGs were obtained from the Human Protein Atlas (HPA) database (https://www.proteinatlas.org/) (Figure 7C). Further comprehensive analysis of paired BC tissue samples demonstrated that GSDMC was significantly upregulated in tumor tissues (Figures 7D–G). Moreover, based on a 5-year follow-up of 41 BC patients from our center, disease-free survival (DFS) analysis stratified by GSDMC expression level indicated that GSDMC may serve as a potential prognostic biomarker (Figure 7H).

## 4 Discussion

The two defining characteristics of cancer include dysregulated proliferative capacity and escape from regulated PCD pathways (Peng et al., 2022). Research on various PCD pathways in BC has been continuously advancing, aiming to comprehensively elucidate the molecular mechanisms underlying BC pathogenesis and identify novel therapeutic targets (Hong et al., 2025). PANoptosis, a newly identified form of PCD proposed 5 years ago, remains poorly understood in terms of its functional role in BC. To further investigate its potential biological functions in BC progression and treatment, this study integrated multi-omics data to construct and validate a prognostic prediction signature based on PANRGs. Notably, this signature not only demonstrated robust prognostic predictive performance but also revealed significant differences in molecular characteristics, TME, and drug sensitivity among different risk groups, thereby providing new theoretical foundations and potential therapeutic targets for precision medicine in BC.

Our study identified five pivotal PANRGs (AIFM1, GSDMC, IL18, IRF1 and GZMB) that play crucial regulatory roles in BC prognosis. Notably, AIFM1 and GSDMC were characterized as high-risk biomarkers, with their elevated expression levels demonstrating a significant correlation with poor clinical outcomes. AIFM1 (Apoptosis-Inducing Factor) was initially identified as a mitochondrial intermembrane space (IMS) protein (Salscheider et al., 2022). When cells undergo apoptosis or damage, AIFM1 is released from mitochondria into the cytoplasm and nucleus, triggering two hallmark caspase-independent apoptotic events: chromatin condensation and large-scale DNA fragmentation (Zhou et al., 2022). Research suggested that AIFM1 expression varies among different cancers, demonstrating a distinct overexpression pattern in BC. Notably, AIFM1 knockdown significantly impaired BC cell proliferation, migration, and invasion (Xu et al., 2025). GSDMC, as a member of the gasdermin family, recent studies have revealed that it mediates pyroptosis in tumor cells through caspase-8/6 activation and its high expression in BC was associated with poor survival prognosis (Hou et al., 2020). IL-18, a key pro-inflammatory cytokine, mediates immune activation by enhancing both innate immunity and adaptive immune responses (Yasuda et al., 2019). As a key downstream effector molecule of the inflammasome-caspase-1 axis, the maturation and release of IL-18 are directly regulated by the PANoptosome complex, thereby promoting a pro-inflammatory microenvironment and synergistically amplifying PANoptosis signaling (Karki and Kanneganti, 2023). IRF1 (Interferon Regulatory Factor 1), originally identified as an interferon regulatory factor, is now widely recognized as a tumor suppressor (Schwartz et al., 2023). Its antiproliferative effects are primarily mediated through the induction of specific target genes that negatively regulate cell growth, including protein kinase R (PKR) and signal transducer and activator of transcription 1 (STAT1) in the JAK-STAT pathway (Alsamman and El-Masry, 2018). Activation of these genes promotes cell cycle arrest or apoptosis, thereby contributing to IRF1’s tumor-suppressive function. IRF1 has recently been recognized as a critical transcriptional regulator of PANoptosis, directly controlling the expression of PANoptosome components such as ZBP1, AIM2, RIPK1, and NLRP12 (Sharma et al., 2023). GZMB (Granzyme B), a serine protease synthesized by natural killer (NK) cells and CD8^+^ T cells, is stored within cytotoxic granules (Gleave and Granville, 2023). Beyond its well-characterized intracellular pro-apoptotic function, GZMB also participates in extracellular matrix (ECM) remodeling and immune regulation when released into the extracellular space (Thompson and Cao, 2024). GZMB directly cleaves GSDME to trigger inflammatory pyroptosis, thereby converting non-inflammatory immune cell-mediated killing into a pro-inflammatory form of cell death that bridges granzyme cytotoxicity with PANoptosis (Zhang Z. et al., 2021). These key PANRGs collectively shape a distinctive prognostic signature for BC. Subsequent validation across multiple independent cohorts consistently demonstrated the superior predictive performance of our model, which showed significant correlations with clinicopathological characteristics (T and M stages). Notably, high-risk patients were more likely to present with advanced T stages (T3-T4) and distant metastases (M1), aligning with previous findings that apoptosis inhibition promotes BC invasion and metastasis (Medina-Ram et al., 2011). The predictive accuracy was further enhanced when the nomogram incorporated both risk scores and age (1-year AUC = 0.816). Particularly noteworthy, the calibration curve maintained good performance even at 3-year predictions, indicating reliable performance for intermediate-to-long-term prognostic assessments.

Our comprehensive multi-algorithmic analysis revealed significant differences in the TME between high- and low-risk groups. The low-risk group exhibited characteristic “hot tumor” features, including abundant T-cell infiltration, high immune scores, and elevated TMB, albeit accompanied by T-cell exhaustion. Notably, IRF1 and GZMB showed strong positive correlations with effector immune cells, suggesting their potential protective role through anti-tumor immune activation. In striking contrast, the high-risk group demonstrated an immunosuppressive TME marked by M2 macrophage enrichment, reduced CD8^+^ T-cell infiltration, and significant immune exclusion. This “cold tumor” phenotype likely underlies the poor response to conventional therapies, particularly immunotherapy (Khosravi et al., 2024). These findings align with established evidence that immune infiltration patterns significantly impact patient outcomes (Savas et al., 2018). TIDE analysis revealed a paradoxical observation: while the low-risk group showed more severe T-cell dysfunction, it exhibited lower immune exclusion and higher MSI scores, suggesting potential sensitivity to PD-1/PD-L1 inhibitors (Wu et al., 2019). Conversely, the high-risk group maintained relatively preserved T-cell function but overall immune paucity, possibly necessitating combinatorial immune activation strategies. These insights provide critical guidance for immunotherapy stratification in BC. Macrophage trajectory analysis revealed a biphasic IL18 expression pattern (initial rise followed by decline, with overall increased expression) coupled with progressive IRF1 downregulation. Building on Choi et al.'s findings that IRF1 inhibition promotes M2 polarization to create an immunosuppressive niche, we postulate that high-risk group tumor-associated macrophages may undergo dynamic phenotypic switching from anti-tumor M1 to pro-tumor M2 states (Zhang et al., 2024; Choi et al., 2024). Beyond macrophages, our single-cell analysis also revealed markedly enhanced fibroblast-tumor cell communications in the high-risk group, characterized by increased interaction strength and elevated signaling of TENASCIN and TGFβ. TGFβ is known to promote cancer-associated fibroblast activation and immune exclusion, while TENASCIN facilitates tumor invasion and metastasis through integrin-mediated pathways (Tauriello et al., 2022; Dhaouadi et al., 2024). These findings suggest that PANoptosis-related genes may coordinately remodel the TME not only by driving macrophage M2 polarization but also by activating fibroblast-mediated pro-tumor signaling, thereby contributing to the immunosuppressive “cold tumor” phenotype in high-risk patients.

Through GSEA analysis, we found that the high-risk group was significantly enriched in metabolism-related pathways, particularly those involved in glucose metabolism, suggesting that tumor cells may undergo glucose metabolic reprogramming to meet the anabolic demands of rapid proliferation (Hay, 2016). This may represent one of the key mechanisms underlying the reduced sensitivity to conventional chemotherapy observed in the high-risk group (Lin et al., 2019). In contrast, the low-risk group was characterized by the activation of immune-related pathways, implying that a favorable TME may contribute to better prognosis. Mutation profiling revealed a higher frequency of TP53 mutations in the high-risk group. TP53 mutations are known to increase lifetime cancer risk and can provide valuable guidance for treatment selection (Schon and Tischkowitz, 2018). The low-risk group exhibited more frequent PIK3CA and CDH1 mutations. Notably, targeted inhibitors against PIK3CA have shown promising clinical results (Turner et al., 2024). These distinct mutation profiles may directly influence the development of individualized treatment strategies. Drug sensitivity analysis provided direct and compelling evidence to guide clinical drug selection. We observed that the high-risk group exhibited reduced sensitivity to many conventional chemotherapeutic agents (such as doxorubicin and gemcitabine) but showed enhanced responses to HER2/mTOR/EGFR-targeted drugs (e.g., tucatinib, everolimus, and gefitinib). This dual-mode response profile has important clinical implications, suggesting that high-risk patients may benefit more from targeted therapies.

GSDMC, a core gene identified in this study, was originally isolated from mouse melanoma cells (Zou et al., 2021). As a member of the gasdermin family, GSDMC has been suggested to modulate pyroptosis, thereby influencing the immune status of the TME and malignant progression (Hou et al., 2020). Compared to other gasdermin members such as GSDMD and GSDME, GSDMC remains relatively understudied (Zhang JY. et al., 2021). Its role in cancer progression is controversial: on one hand, GSDMC is highly expressed in pancreatic cancer and promotes tumor progression by upregulating genes associated with stemness, EMT, and immune evasion (Wu et al., 2024). Silencing GSDMC in colorectal cancer cell lines suppressed tumor proliferation, while its overexpression enhanced proliferative capacity (Miguchi et al., 2016), supporting an oncogenic role. On the other hand, GSDMC has been reported to act as a tumor suppressor in gastric and esophageal carcinogenesis (Saeki et al., 2009).

Our study demonstrated that GSDMC was significantly upregulated in BC tissues. Its high expression was closely associated with poor patient prognosis. These findings suggest considerable clinical relevance. However, its precise biological mechanisms remain to be fully elucidated. It is noteworthy that high GSDMC expression in the high-risk group was accompanied by an immunosuppressive TME, implying potential involvement in immune evasion or metabolic reprogramming. Future studies should focus on elucidating the upstream regulatory mechanisms and downstream signaling networks of GSDMC in BC, particularly its interactions with non-coding RNAs and post-translational modifications. Importantly, our drug sensitivity analysis indicated that groups with high GSDMC expression showed increased sensitivity to multiple targeted agents, suggesting its potential role as a predictive biomarker for treatment response. These findings provide new insights into the complex functions of GSDMC in BC and lay a theoretical foundation for the development of anti-cancer strategies targeting the gasdermin family.

## 5 Conclusion

This study established a robust prognostic model for BC based on PANRGs, which demonstrates consistent predictive performance. The integrated multi-omics characteristics provide new insights into the heterogeneity of BC. The differences in TME, metabolic profiles, and drug sensitivity between high- and low-risk groups offer a theoretical foundation for precision medicine. In particular, high-risk patients may benefit from targeted therapeutic strategies. The identification of key genes such as GSDMC suggests promising novel targets for BC treatment. Together, these findings may facilitate the advancement of individualized diagnosis and treatment for BC patients.

## Data Availability

The raw data supporting the conclusions of this article will be made available by the authors, without undue reservation.
