# Supplementary material for: Integrative PANoptosis-focused omics analysis uncovers GSDMC as a candidate biomarker in breast cancer
Source: Front Cell Dev Biol. 2026 Jun 15;14:1841350. doi: 10.3389/fcell.2026.1841350 (PMC13311090; doi:10.3389/fcell.2026.1841350)
Supplement: Supplementary file 1 [file Supplementaryfile1.docx]

Supplementary Material

## Supplementary Figures

**
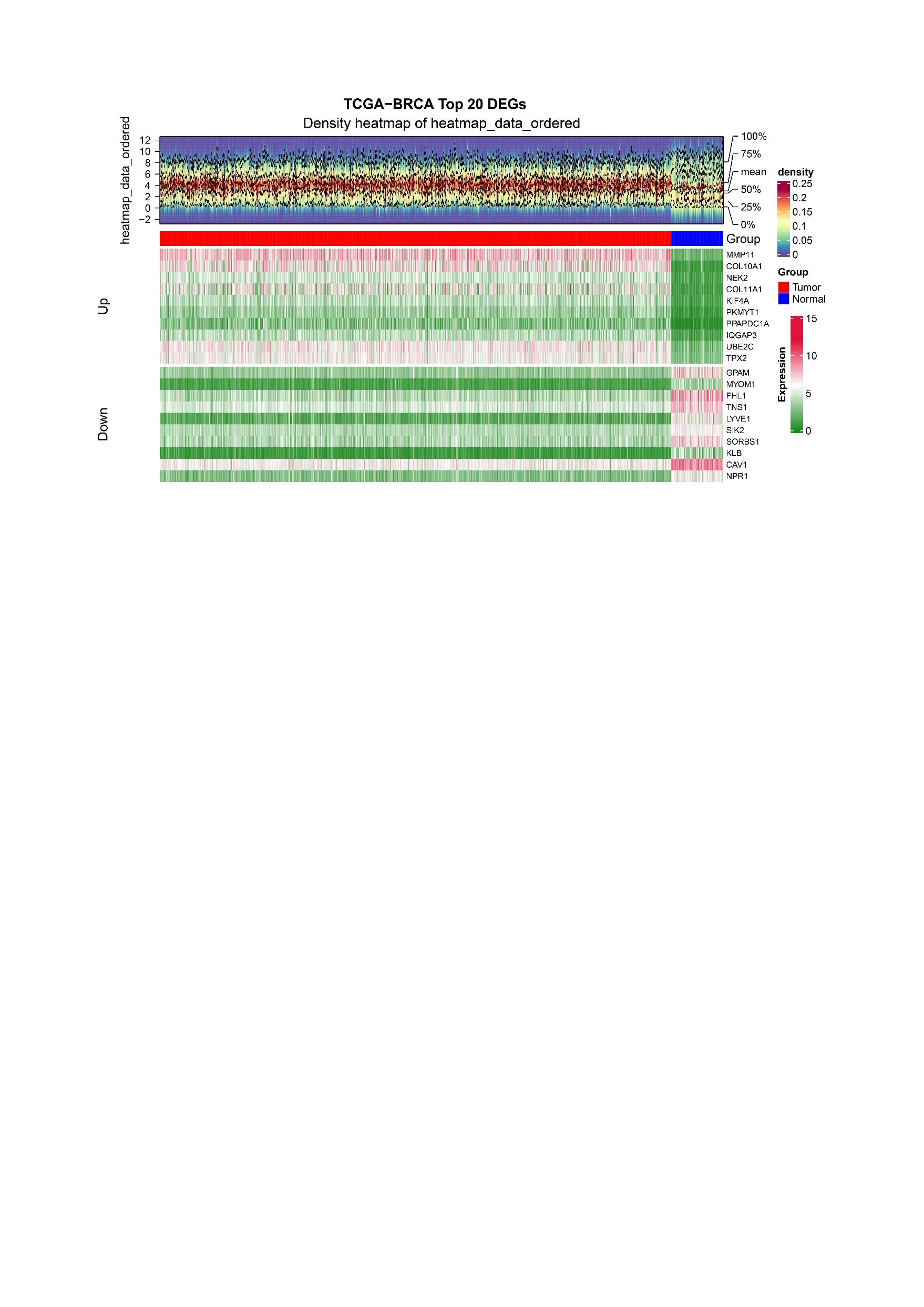
**

**Supplementary Figure 1.** Heatmap of the top 20 DEGs.


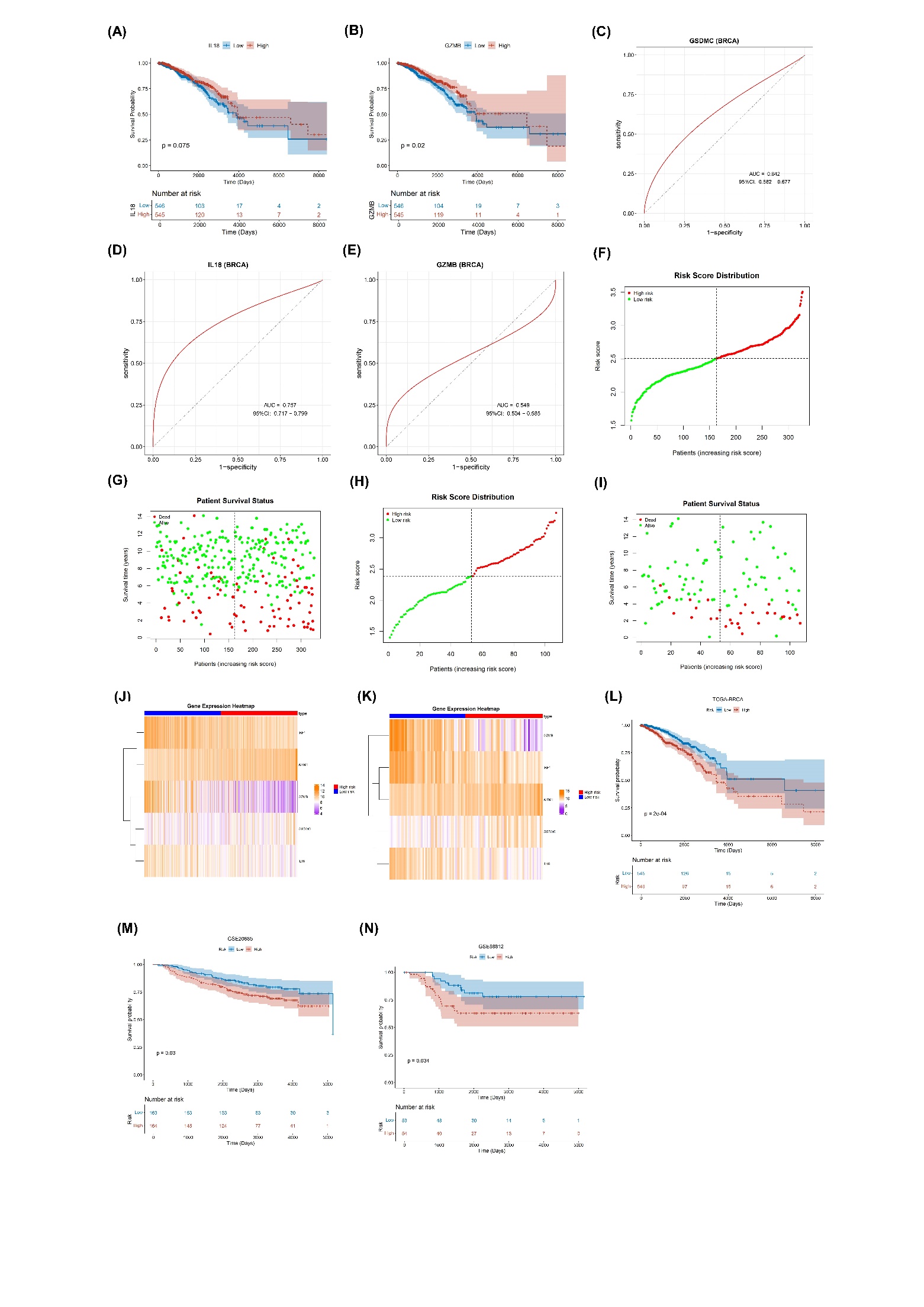


**Supplementary Figure 2. Validation of risk signatures for breast cancer patients based on the TCGA-BRCA database.**

(A-B) Kaplan-Meier overall survival curves comparing groups based on the expression of the PANRGs; (C-E) ROC curve analysis of PANRGs for discriminating between tumor and normal tissue; (F) Distribution of PANRG risk scores in the GSE20685 validation set; (G) OS status distribution by risk group in GSE20685; (H) PANRG risk score distribution in the GSE55812 validation set; (I) OS status distribution by risk group in GSE55812; (J) Expression of the 5 PANRGs across risk groups in GSE20685; (K) PANRG expression patterns across risk groups in GSE55812; (L-N) Kaplan-Meier overall survival curves comparing risk groups (TCGA-BRCA cohort, GSE20685 and GSE55812 validation sets).


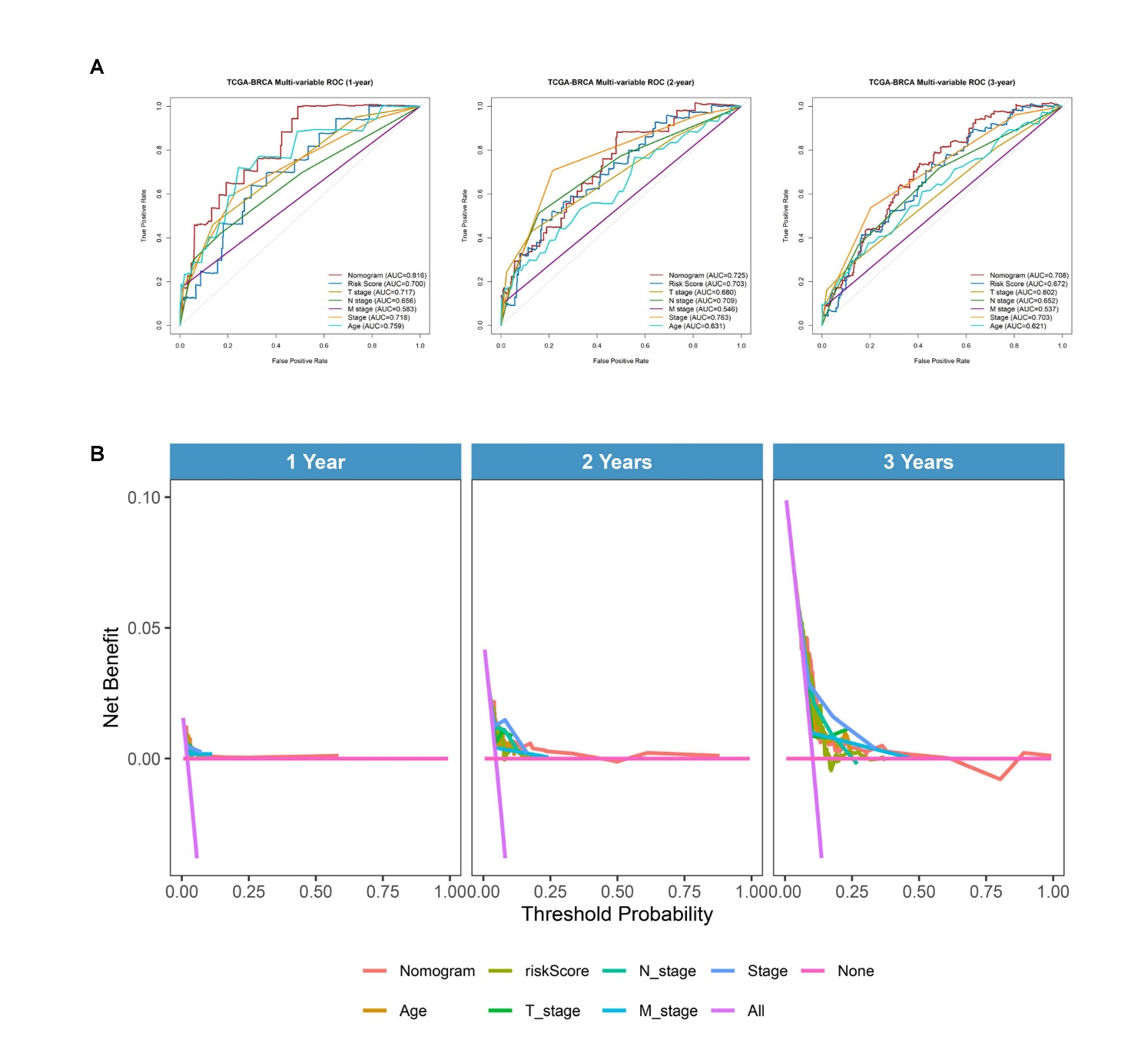


**Supplementary Figure 3.**

1. ROC curve of the nomogram prediction model; (B) DCA of the nomogram prediction model.


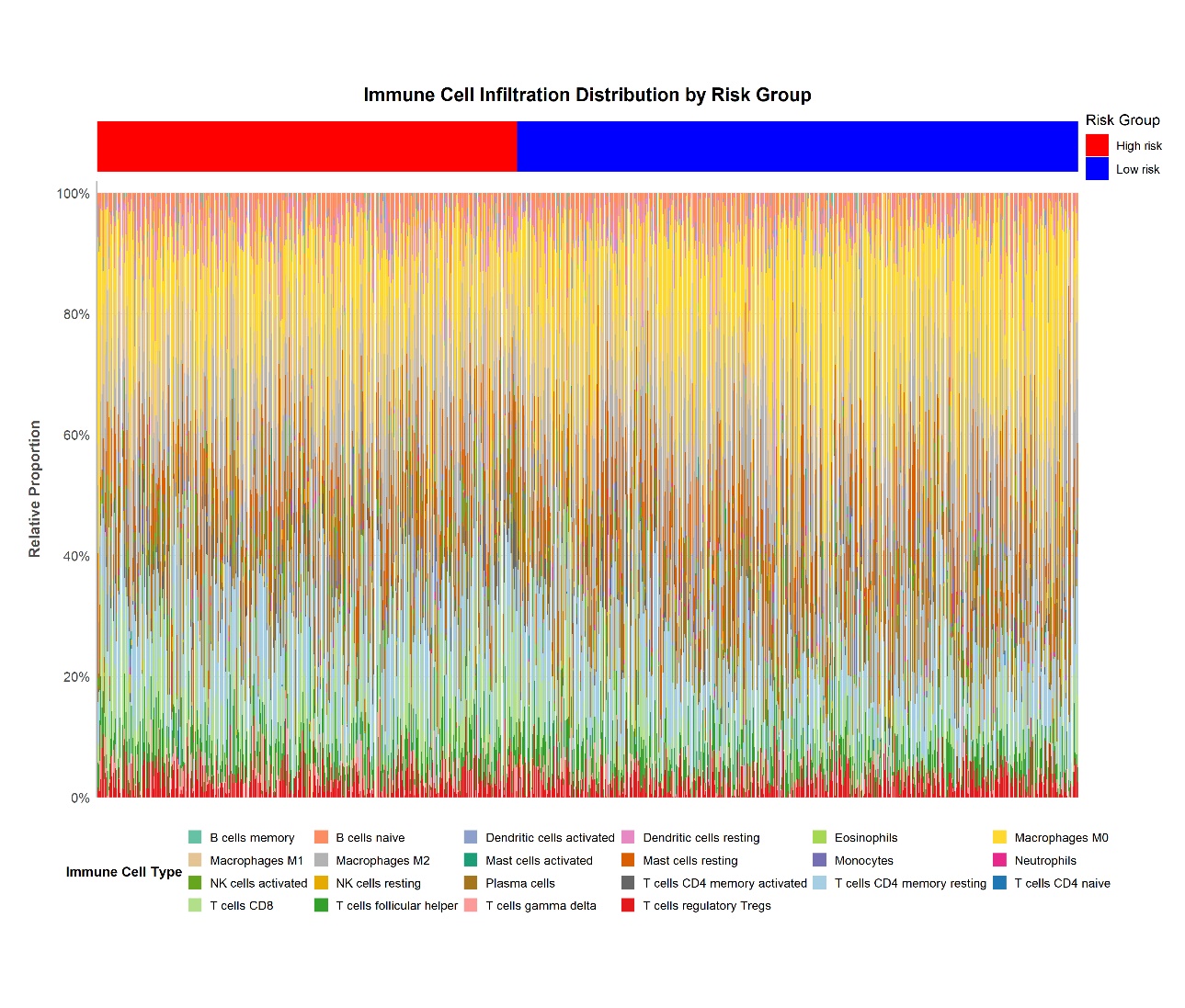


**Supplementary Figure 4. Distribution of immune cells in different risk groups.**


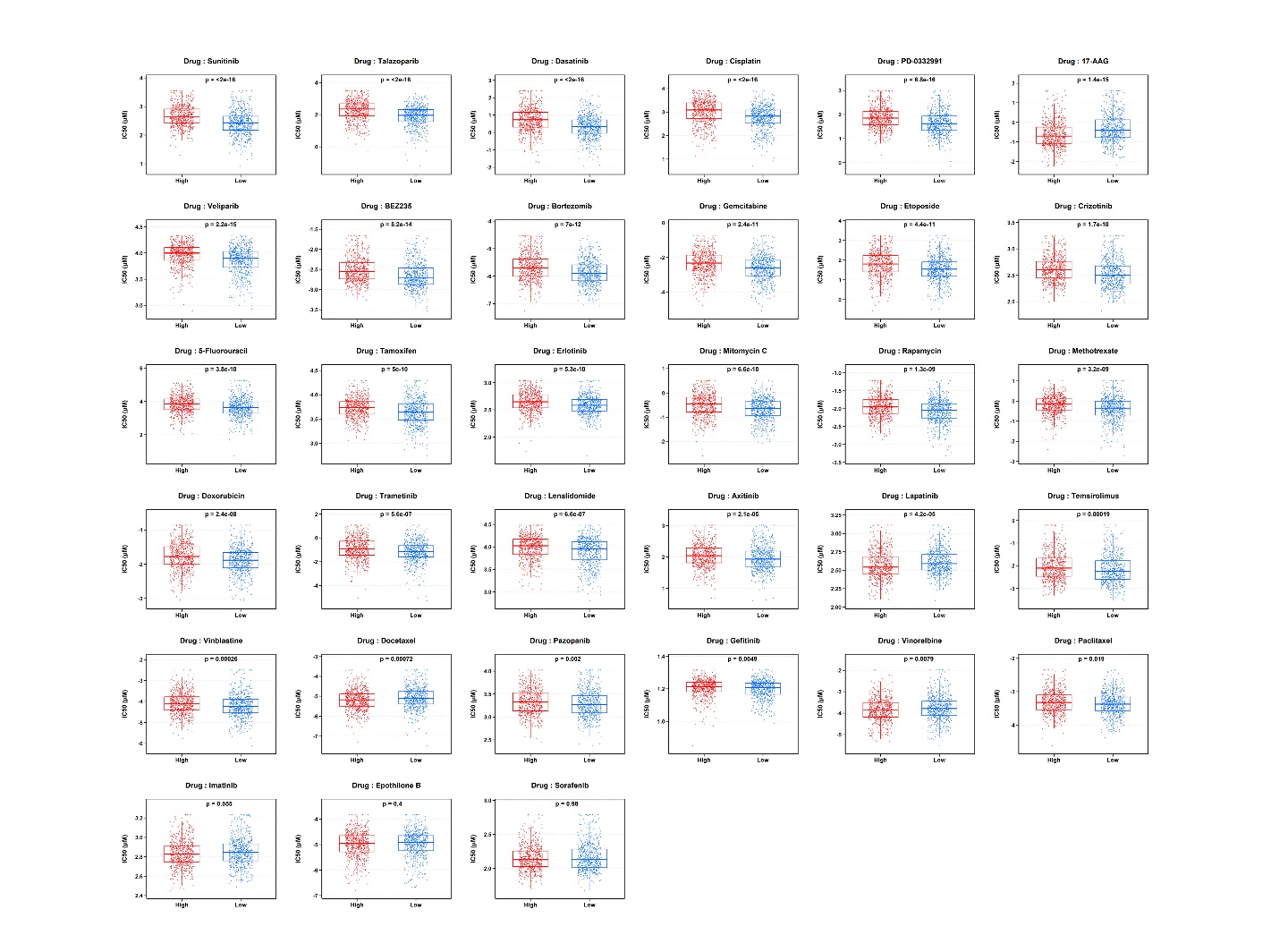


**Supplementary Figure 5. IC50 values for 33 frequently used breast cancer drugs from the GDSC database.**


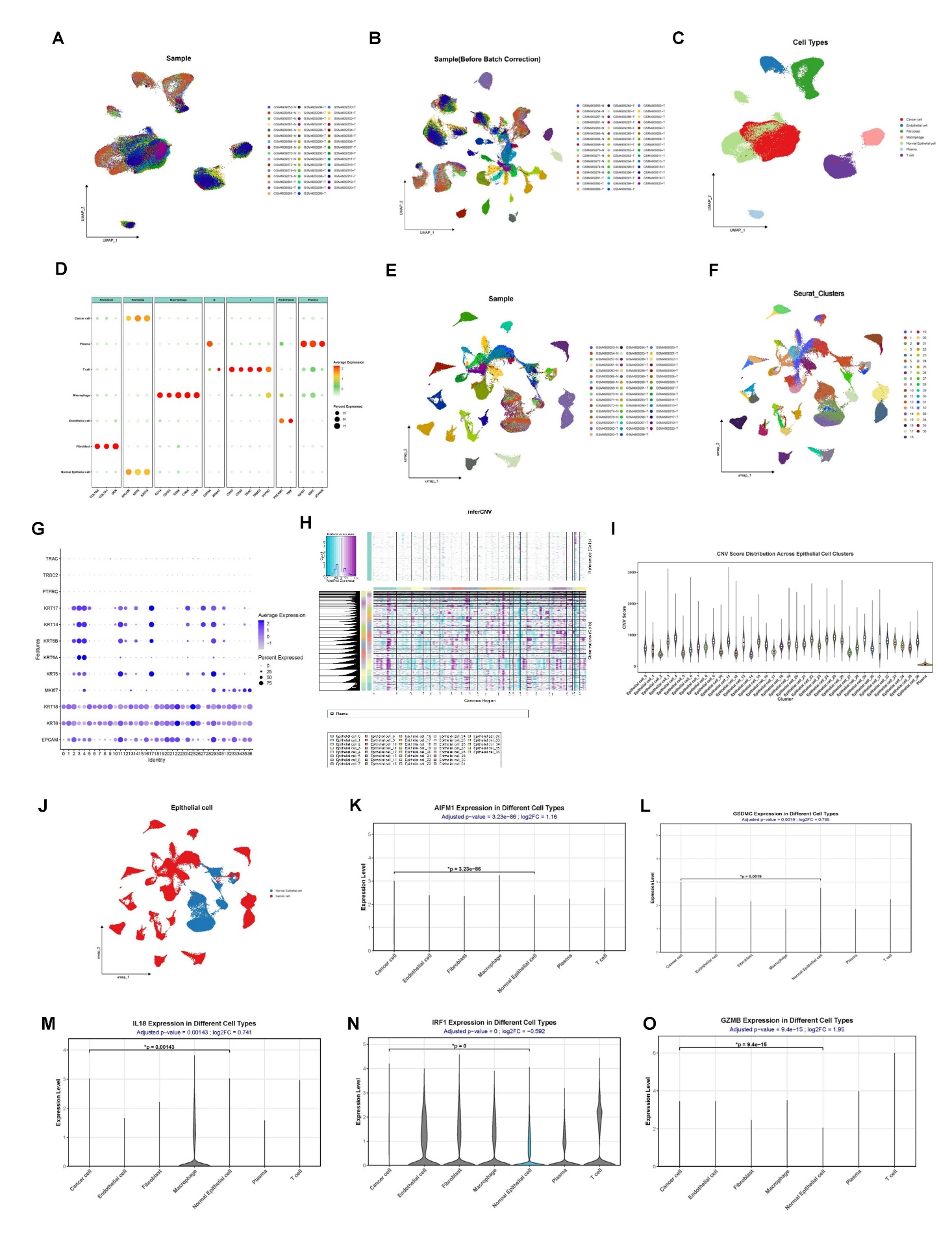


**Supplementary Figure 6. Identification of epithelial subpopulations and CNV analysis in the TME via single-cell RNA sequencing.**

(A)UMAP visualization of integrated multi-sample scRNA-seq data (GSE161529 dataset); (B) UMAP visualization of integrated multi-sample scRNA-seq data before Batch Correction; (C)UMAP plot of cell clusters; (D)Cell type annotation based on canonical marker genes; (E)UMAP plot of integrated multi-sample epithelial cells; (F)UMAP plot of reclustered epithelial cells (37 subpopulations); (G)Bubble plot confirming the exclusion of non-epithelial cell contamination (based on marker gene expression); (H)Heatmap of chromosomal CNVs; (I) CNV score in epithelial subpopulations; J) CNV-based classification of normal (blue) vs. malignant (red) epithelial cells; (K-O) The landscape of AIFM1, GSDMC, IRF1, IL18 and GZMB expression distribution among various cell populations in the breast cancer microenvironment.
